# Anterior knee pain and its intrinsic risk factors among runners in under-resourced communities in Ekurhuleni, Gauteng

**DOI:** 10.4102/sajp.v74i1.452

**Published:** 2018-09-13

**Authors:** Siyabonga H. Kunene, Serela Ramklass, Nomathemba P. Taukobong

**Affiliations:** 1 Department of Physiotherapy, University of the Witwatersrand, South Africa; 2 School of Clinical Medicine, University of KwaZulu-Natal, South Africa; 3 Department of Institutional Planning, Sefako Makgatho Health Sciences University, South Africa

## Abstract

**Background:**

Anterior knee pain (AKP) is the most common injury among runners, especially in females and young runners. Because of a deficit of rehabilitation services in under-resourced communities, runners train and compete with injuries, resulting in further complications.

**Objectives:**

This study aimed to determine the prevalence and modifiable intrinsic risk factors for AKP among runners in under-resourced communities in Ekurhuleni, Gauteng Province.

**Method:**

This cross-sectional study included a population of 347 runners from six running clubs. Convenience sampling was used to recruit 183 participants aged between 13 and 55. A standardised questionnaire determined AKP prevalence, and 12 physical tests screened for modifiable intrinsic risk factors. Descriptive and inferential statistical tests were used to analyse the data.

**Results:**

AKP was present in 40% of participants, particularly in males (57.9%) and young runners (57.9%) with 3–5 years of running experience (31.1%). Anterior knee pain was significantly associated with age (chi-square [*χ*^2^] = 6.484, *p* = 0.039) and running experience (*χ*^2^ = 8.389, *p* = 0.036). The modifiable intrinsic risk factors found to have contributed significantly to AKP were: tight hamstrings (odds ratio [OR] = 1.021; *p* = 0.051); tight iliotibial band (OR = 1.1; *p* = 0.046); weak quadriceps (OR = 0.15; *p* = 0.040); weak hip muscles (OR = 1.13; *p* = 0.004) and patellar tilt abnormalities (OR = 1.33; *p* = 0.015).

**Conclusion:**

Anterior knee pain and various modifiable intrinsic risk factors were found among these runners. These findings suggest that management of AKP should take into consideration the effect of these identified modifiable risk factors to improve management outcomes. A community-based rehabilitation approach should be considered, given the lack of resources in low socio-economic communities.

**Clinical implications:**

The results from this study make explicit the risk factors associated with AKP, particularly in runners from under-resourced communities. These are important considerations in the development of rehabilitation programmes to manage AKP.

## Introduction

Running is a popular, low-cost sport that is enjoyed by people of all ages worldwide. Despite its health benefits, runners experience injuries during training and/or competition that negatively affect their health and performance. The incidence of running injuries for the lower limbs ranges from 19.4% to 79.3% worldwide, with the knee being the most predominant site (Van Gent et al. 2007). The most common symptom of overuse knee injury among runners is anterior knee pain (AKP), which is commonly caused by patellofemoral pain and patellar tendinopathy (Brukner & Khan 2013). Other less common causes may include fat pad impingement, synovial plica, prepatellar bursitis, quadriceps tendinopathy, infrapatellar bursitis and patellofemoral instability (Brukner & Khan 2013). However, the causes of AKP remain controversial and unclear, with little supporting empirical evidence (Brukner & Khan 2013).

The prevalence of AKP ranges between 15% and 45% globally (Cook et al. 2010). AKP mostly affects females, adolescents and younger adults (Boling et al. 2010; Brukner & Khan 2013; Van Gent et al. 2007). It is described as deep intermittent pain on or around the margins of the patella. Other clinical features may include crepitus, clicking within the knee and a feeling of instability in the joint (Brukner & Khan 2013). These clinical features are experienced during activities such as running, squatting, going up and down stairs, cycling and jumping (Witvrouw et al. 2000).

Various intrinsic and extrinsic risk factors have been identified that contribute to AKP (Waryasz & McDermott 2008). Extrinsic risk factors include the type of activity, manner in which the activity is performed, environmental conditions and the effect of the equipment used during physical activity, for example air resistance, gravity and ground reaction forces, and shoes (Brukner & Khan 2013). Intrinsic risk factors relate to individual physical characteristics and psychological traits (Brukner & Khan 2013). The intrinsic factors include quadriceps weakness (especially in the vastus medialis oblique [VMO]), tightness of the gastrocnemius–soleus complex, dysfunction of the hip muscles, foot overpronation, generalised joint laxity, limb length discrepancy, patellar malalignment and patellar hypermobility (Brukner & Khan 2013; Halabchi, Mazaheri & Seif-Barghi 2013).

The first author has observed that injuries associated with AKP are experienced by many runners from under-resourced communities and often unnecessarily spell the end of their running careers because of the lack of rehabilitation services in these communities. Although AKP responds to conservative management in 95% of cases (Tria, Palumbo & Alicea 1992), poor rehabilitation services lead to runners training and competing with injuries, which results in further complications. It is widely recognised that athletes from low socio-economic communities generally present with poorer health outcomes than those from better resourced communities (Finch et al. 2008; Golle et al. 2014). According to Finch et al. (2008), athletes from low socio-economic communities present with increased risk of injuries because of various factors including the lack of health resources, coaching or training professionals, people to organise and deliver sports, and attitudes towards injuries.

There has not been a study in South Africa that has reported on AKP prevalence and its risk factors in poorly resourced communities. Hence the aim of this study was to determine the prevalence and modifiable intrinsic risk factors for AKP among runners in under-resourced peri-urban communities in Ekurhuleni, Gauteng Province.

## Methods

This cross-sectional study population included 347 long-distance recreational runners from six developmental running clubs, in under-resourced peri-urban communities in Ekurhuleni, Gauteng Province. A Raosoft statistical tool was used to calculate a sample size of 183 participants, taking into consideration a 95% confidence level, 5% margin of error and 50% response distribution. Participants included runners aged between 13 and 55 years, conveniently recruited during their training sessions at the various training grounds.

### Measuring instruments

Self-administered questionnaires were used to collect demographic and AKP-related data. Questionnaire 1 comprised demographic questions, which included gender, age, race, running experience, hand dominance, height and weight. Questionnaire 2 was the standardised AKP questionnaire by Kujala et al. (1993), which consists of 13 short questions that assessed the participants’ knee symptoms and their functional limitations associated with AKP. The standardised AKP questionnaire has good test–retest reliability (intraclass correlation coefficient [ICC] = 0.92) and validity (Kujala et al. 1993; Singer & Singer 2009).

Modifiable intrinsic risk factors were screened using 12 physical tests as follows:

The one-legged hop test assessed weakness of the quadriceps muscle, especially the vastus medialis. This test was performed three times with each leg and the hop distance was measured in centimetres from the toe. A quotient (%) between two legs was registered and was defined as abnormal if the quotient was less than 85%. The one-legged hop test has high reliability (ICC = 0.96) (Ageberg, Zätterström & Moritz 1998).

The passive knee extension test assessed hamstring muscle tightness. In this test, the examiner positioned the athlete supine and passively lifted the straightened leg and measured hip flexion. The test is considered positive if the range measured is less than 80° for men and 90° for women. This test has excellent inter-rater reliability (ICC = 0.93) and test–retest reliability (ICC = 0.94–0.96) (Gabbe et al. 2004).

To measure tightness of iliopsoas muscles, a modified Thomas test was used. The participant perched on the end of the plinth and rolled back to supine while holding both knees with both hands close to the chest. He or she was then asked to release one leg and hold the other one in full hip flexion. The examiner then assessed the hip flexion angle of the released leg. If the angle was greater than zero (above the horizontal line), the test was considered positive, which indicated a shortened iliopsoas muscles. This test has demonstrated very good to excellent inter-rater reliability (ICC = 0.92) and test–retest reliability (ICC = 0.63–0.75) (Gabbe et al. 2004).

The Ober test measured the tightness of the iliotibial band muscle. This test was done while side lying. The lower leg of the participant was flexed to 45° knee flexion while the examiner stabilised the pelvis. The top knee was flexed to 90° and the hip was then passively brought to abduction and extension. The examiner then gently released the leg into adduction. If the hip remained abducted (above horizontal level) the test was considered positive. The intratester and intertester reliability of this test are very good (ICC = 0.94 and 0.73, respectively) (Melchione & Sullivan 1993).

The weight-bearing lunge test measured tightness of the gastrocsoleus complex. This test measures the angle of dorsiflexion at the ankle joint. The participant placed the tested foot forward on the floor and was asked to lunge forward and try to touch the wall in front with his or her knee while the other leg was placed backwards. The distance between the toe and wall was gradually increased until no further ankle range was achieved. The distance between the big toe and the wall was then measured using a tape measure. A distance of less than 12.5 cm indicated a positive test. The weight-bearing lunge test has been found to have excellent intrarater (ICC = 0.97–0.98) and inter-rater (ICC = 0.99) reliability (Bennell et al. 1998).

To measure hip muscle dysfunction (abductor muscle), a Trendelenburg test was used. In this test the participant stood on one leg with both hands on the hips and the examiner observed hip alignment (both hips maintained at a horizontal level). If during unilateral weight bearing the pelvis dropped towards the unsupported side, the test was considered positive. The Trendelenburg test has been used successfully by various authors to assess hip abductor muscle dysfunction (Asayama et al. 2002; Baker & Bitoums 1986; Barber et al. 1996; Downing et al. 2001; Inan et al. 2005; Pai 1996; Ramesh et al. 1996; Reikeraas et al. 1996).

The Foot Posture Index version 6 (FPI-6) measures foot pronation abnormalities. This is a six-item assessment tool with each item scoring between –2 and +2 to give a sum of –12 (highly supinated) and +12 (highly pronated). The following are the items assessed when using this method: talar head palpation, curve above and below the lateral malleoli, calcaneal angle, talonavicular bulge, medial longitudinal arch and forefoot-to-rearfoot alignment. The FPI-6 tool has demonstrated good inter-item reliability (Cronbach’s alpha = 0.83) (Redmond, Crosbie & Ouvrier 2006).

To assess for general joint laxity, a Beighon and Haraan Joint Mobility Index (BHJMI) was used. This tool has a scoring of 0–9, where the higher score indicates greater joint laxity. Mobility of the little fingers, thumbs, elbows, knees and trunk forward flexion are assessed. The BHJMI has been shown to have good inter-rater reliability (ICC = 0.82) and intrarater reliability (ICC = 0.92) (Boyle, Witt & Riegger-Krugh 2003).

A tape measure was used to assess limb length discrepancy. Leg length differences were evaluated by measuring the distance between the anterior superior iliac spine and the medial malleolus of both legs in supine. This test has high intrarater and inter-rater reliability (ICC = 0.89–0.99) (Gogia & Braatz 1986; Hoyle, Latour & Bohannon 1991).

Patellar tilt and mediolateral glide tests were performed in order to assess patellar malalignment. The patellar tilt test was performed by assessing the height of the medial patella border with that of the lateral patellar border. The examiner placed his thumb and index finger on the medial and lateral border of the patella. If the digit palpating the medial border was more anterior than the lateral border, then the patella was tilted laterally. If the digit palpating the lateral border was more anterior than the medial border, then the patella was tilted medially (Dixit et al. 2007). The mediolateral glide test was also performed using a tape measure to record the distance from the mid-patella to the lateral femoral epicondyle and the distance from the mid-patella to the medial femoral epicondyle. Normal alignment was indicated by the position of the patellar equidistant from each epicondyle. These tests have been used successfully in other studies; however, the intrarater and inter-rater reliability has been found to be low (ICC = 0.44–0.50 and 0.20–0.35, respectively) (Watson et al. 2001).

The patellar mobility test was used to measure the passive mediolateral range of resting patellar motion and the integrity and tightness of the medial and lateral restraints. The test was performed with the participant’s knee flexed 20° – 30° and the quadriceps muscle completely relaxed. Lateral patellar mobility of three quadrants is suggested as incompetent medial restraint, and medial mobility of three quadrants suggests patella hypermobility (Dixit et al. 2007). The patellar mobility test has been used successfully by various authors, but its reliability has been found to be quite low. Intrarater reliability varies from 0.39 to 0.47 and inter-rater reliability is 0.31 (Watson et al. 2001).

### Data collection procedure

Runners were given leaflets containing the purpose, objectives and methods of the study and were requested to complete consent forms if they wished to participate in the study. Consent was obtained from parents or guardians of participants younger than 18 years and assent from these participants. A pilot study was conducted among 18 participants prior to the main study and no adjustments were required on the data collection tools used; hence, the data obtained were included in the main study. The first author distributed questionnaires to be completed by participants during their training sessions and then conducted the screening tests immediately after the participants completed the questionnaires. Screening data were captured on a data collection sheet developed by the first author.

### Data analyses

Data were captured in Microsoft Excel and then imported into SPSS for analysis. Descriptive and inferential statistics were used to analyse data. Descriptive statistics included the calculation of frequencies and percentages. Inferential statistics included chi-square tests, odds ratios (OR) and a logistic regression used to determine associated intrinsic factors. The level of significance was set at *p* = 0.05.

### Ethical considerations

Ethical clearance for this study was obtained from the University of KwaZulu-Natal (clearance certificate no. BFC377/15).

## Results

All 183 participants completed the questionnaires and participated in the screening. As indicated in Table 1, the majority of participants were black (48, 80.9%), male (106, 57.9%), mostly young (18–35 years old; 94, 51.4%) with 3–5 years of running experience (57, 31.1%). Most participants presented with a normal body mass index (110, 60.1%).

**TABLE 1:** Demographics profile (*n* = 183).

Demographics	Categories	Frequency (*n*)	Percentage (%)
Gender	Male	106	57.9
	Female	77	42.1
Age	13–17	51	27.9
	18–35	94	51.4
	36–55	38	20.8
Race	White	01	0.5
	Black	148	80.9
	Mixed race	34	18.6
Hand dominance	Left	31	17.0
	Right	152	83.0
Running experience	< 1 year	20	10.9
	1–3 years	49	26.8
	3–5 years	57	31.1
	6–10 years	37	20.2
	> 10 years	20	10.9
Body mass index (BMI)	< 18.5	28	15.3
	18.5–24.9	110	60.1
	25–29.9	42	23.0
	> 30	03	1.6

**FIGURE 1:**
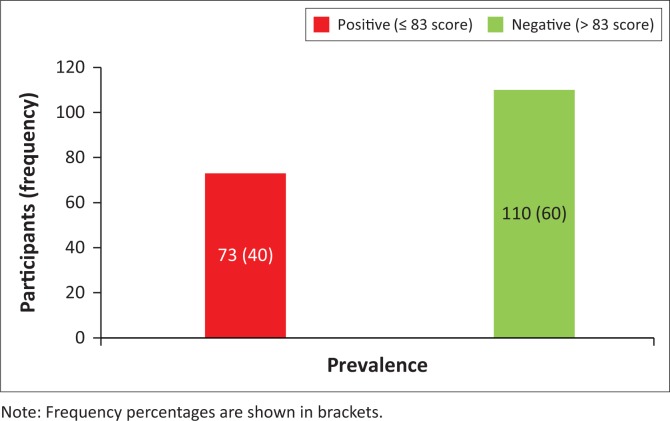
Prevalence of anterior knee pain (*n* = 183).

Anterior knee pain was present in 73 (40%) participants (those who presented with a score of ≤ 83 according to the standardised AKP questionnaire used) (Figure 1). A prevalence of AKP of 33 (18%) was noted among participants between the ages of 18 and 35 years (Table 2).

**TABLE 2:** Anterior knee pain and demographic profile (*n* = 183).

Demographics	Categories	Anterior knee pain		Chi-square (*χ*^2^)	*p*
		Frequency (*n*)	Percentage (%)		
Gender	Male	40	21.9	0.488	0.485
	Female	33	18.0		
Age	13–17	18	9.8	6.484	0.039
	18–35	33	18.0		
	36–55	22	12.0		
Race	White	1	0.5	3.134	0.371
	Black	61	33.3		
	Mixed race	11	6.0		
Hand dominance	Left	9	4.9	1.835	0.176
	Right	64	35.0		
Running experience	< 1 year	7	4.8	8.389	0.036
	1–3 years	22	12.0		
	3–5 years	19	10.4		
	6–10 years	12	6.6		
	> 10 years	13	7.1		
Body mass index (BMI)	< 18.5	12	6.6	5.377	0.146
	18.5–24.9	44	24.0		
	25–29.9	14	7.7		
	> 30	3	1.6		

Anterior knee pain was strongly associated with age (*χ*^2^ = 6.484, *p* = 0.039) and was also strongly associated with running experience (*χ*^2^ = 8.389, *p* = 0.036), where most affected participants had running experience of 3–5 years (10.4%). Anterior knee pain was not associated with gender (*χ*^2^ = 0.488, *p* = 0.485), race (*χ*^2^ = 3.134, *p* = 0.371), hand dominance (*χ*^2^ = 1.835, *p* = 0.176) or BMI (*χ*^2^ = 5.377, *p* = 0.146) (Table 2).

The binary logistic regression of risk factors for AKP among the participants is represented in Table 3. The model used explained 33% (Nagelkerke *R*^2^) of the variance in AKP and correctly classified 88.3% of cases. Modifiable intrinsic risk factors for AKP were found in most participants. The following factors contributed significantly to AKP: tightness of hamstrings (OR = 1.021; *p* = 0.051); tightness of iliotibial band (OR = 1.122; *p* = 0.046); quadriceps muscle weakness (OR = 0.154; *p* = 0.040); hip control muscle weakness (OR = 1.131; *p* = 0.004) and patellar tilt abnormalities (OR = 1.332; *p* = 0.015).

**TABLE 3:** Logistic regression of modifiable intrinsic risk factors (*n* = 183).

Risk factors	Category	Frequency (*n*)	Percentage (%)	*p*	Odds ratio	95% confidence interval
Quadriceps muscle malfunction	Left	17	9.0	-	1	-
	Right	17	9.0	0.765	0.244	0.118–1.200
	Both	43	18.0	0.040	0.154	0.111–1.620
Hamstring muscle tightness	Left	7	4.0	-	1	-
	Right	8	4.0	0.077	0.453	0.163–1.251
	Both	77	42.0	0.051	1.021	1.010–2.197
Iliopsoas muscle tightness	Left	6	3.0	-	1	-
	Right	7	4.0	0.994	0.316	0.006–1.814
	Both	173	95.0	0.152	1.023	1.022–2.926
Iliotibial band muscle tightness	Left	22	12.0	-	1	-
	Right	17	9.0	0.810	1.237	0.218–1.022
	Both	122	67.0	0.046	1.122	1.112–1.565
Gastrocnemius complex tightness	Left	1	0.5	-	1	-
	Right	2	1.0	0.999	0.021	0.001–1.456
	Both	150	82.0	0.084	0.346	0.104–1.152
Hip muscle dysfunction	Left	14	8.0	-	1	-
	Right	20	11.0	0.150	0.307	0.022–1.848
	Both	63	34.0	0.004	1.131	1.112–2.476
Foot pronation	Mild	45	25.0	-	1	-
	Moderate	21	11.0	0.178	0.177	0.026–1.213
	Severe	2	7.0	0.110	1.011	1.002–1.756
General joint laxity	Mild	13	7.0	-	1	-
	Moderate	9	5.0	0.450	1.794	1.394–2.172
	Severe	6	3.0	0.154	1.001	0.998–1.228
Leg length discrepancy	Normal	161	88.0	-	1	-
	Discrepancy	22	12.0	0.123	1.911	1.749–2.320
Patellar tilt abnormalities	Left	22	12.0	-	1	-
	Right	15	8.0	0.893	1.127	0.198–1.398
	Both	46	25.0	0.015	1.332	1.329–2.116
Patellar glide abnormalities	Left	13	7.0	-	1	-
	Right	13	7.0	0.103	0.688	0.768–1.715
	Both	54	29.0	0.797	0.837	0.216–1.241
Patellar hypermobility	Left	17	9.0	-	1	-
	Right	20	11.0	0.168	0.246	0.033–1.804
	Both	63	34.0	0.855	0.899	0.285–1.835

## Discussion

Runners in this study were from communities with scarce rehabilitation services, and this places runners at risk of training and competing with injuries that are not appropriately rehabilitated. From the first author’s experience as a physiotherapist, some runners have missed opportunities to grow in their careers because of injuries that could have been prevented, treated or rehabilitated if physiotherapists had been easily available, accessible and affordable. Anterior knee pain in addition affects the quality of life of athletes in a negative way if not managed well (Cheung, Zhang & Ngai 2013).

Athletes with AKP usually present with pain during walking, running, squatting, going up and down stairs, jumping and pain after prolonged sitting with the knee in flexion (Finch et al. 2008; Waryasz & McDermott 2008; Witvrouw et al. 2000). Furthermore, they may also present with knee swelling, abnormal patella movements, atrophy of thigh muscles and knee flexion deficiency. All these symptoms may contribute to a runner reducing or even stopping their running activity. The AKP prevalence reported in this study shows a high rate (40%), which is at the top end of the commonly cited prevalence of 15% – 45% among the general population (Cook et al. 2010). This high prevalence of AKP among runners could be a result of a lack of healthcare services in these communities. The risk factor findings in this study may also provide an explanation as to the causes of AKP.

Age and running experience were significantly associated with AKP, meaning that younger and less experienced runners were more likely to experience AKP compared to older and more experienced runners. The evidence shows that AKP, in most instances, affects young and inexperienced runners, which include adolescents and young adults (Boling et al. 2010; Brukner & Khan 2013; Van Gent et al. 2007). Anatomical and biomechanical factors may contribute to AKP among young runners but the common underlying reason is mostly overuse injuries. Most young and inexperienced runners are usually affected because of sudden increases in the intensity, duration and volume of running activity; inadequate sport-specific training; poor training techniques and inappropriate equipment used for the sport (Patel & Villalobos 2017). If professional services are scarce in communities (including the communities in this study), injuries and risk of injuries are bound to increase, which may explain why the prevalence of AKP was so high in this study.

There were a variety of modifiable intrinsic risk factors for AKP, which are also suggested in the literature (Halabchi et al. 2013; Witvrouw et al. 2000). Quadriceps and gluteus muscle weakness, hamstring and iliotibial band tightness and patellar malalignment were found to have contributed significantly to AKP compared to the other risk factors. VMO muscle weakness contributes significantly to AKP, which is similar to the findings in our study (Waryasz & McDermott 2008; Witvrouw et al. 2000). The VMO muscle is the main active medial stabiliser of the patella, and the lateral forces caused by the vastus lateralis muscle, lateral retinaculum and iliotibial tract frequently overpower it, if it is weak. There is usually delayed activation of the VMO compared to the vastus lateralis, which can cause an imbalance of forces, which may affect the knee. Restoring VMO strength and overall quadriceps muscle function is therefore necessary.

Dysfunction of the hip muscles (gluteus muscles), especially those responsible for hip abduction and external rotation, contribute to AKP because of their role in maintaining the optimal alignment of the lower limb during weight-bearing related activities (Brukner & Khan 2013; Halabchi et al. 2013). Our study showed that hip abductor and external rotators were significantly weaker for participants with AKP. Similar studies have also shown significantly weaker hip abductors and external rotators (*p* = 0.001–0.049) (Cichanowski et al. 2007; Ireland et al. 2003), although Piva, Goodnite and Childs’ (2005) results are contrary to our study, showing no significant relationship between hip abductors and external rotator muscles and AKP (*p* = 0.218). Our study clearly indicates a need to address hip abductor and external rotator muscle strength in order to prevent or rehabilitate AKP among runners. Therefore, closed and open kinematic chain strengthening exercises are recommended to improve the strength of weak muscles, in this case, VMO and gluteus muscles (Halabchi et al. 2013).

Piva et al. (2005) reported findings similar to our study that showed a strong relationship between AKP and reduced flexibility of the hamstring muscles. They also found a strong relationship between AKP and the tightness of other muscles, including the gastrocnemius (*p* = 0.001), soleus (*p* = 0.001) and quadriceps (*p* = 0.001), which was not the case in our study. Puniello (1993) and Hudson and Darthuy (2009) similarly found that AKP was significantly associated with tightness of the iliotibial band (*p* < 0.005). Therefore, stretching exercises to improve flexibility of tight muscles are crucial and should be included in prehabilitation and rehabilitation programmes to reduce the risks of AKP among runners (Halabchi et al. 2013).

Patellar malalignment is commonly believed to be the primary intrinsic risk factor for AKP (Halabchi et al. 2013), and lateral malalignment or maltracking is an important feature of AKP (Halabchi et al. 2013). Our study showed a significant relationship between AKP and patellar tilt abnormalities (*p* = 0.015), as have Hunter et al. (2007) and Barton et al. (2010). Strapping (using Grelsamer and McConnell’s technique) can be used to treat patellar malalignment and reduce knee pain (Brukner & Khan 2013; Halabchi et al. 2013).

Generalised ligamentous laxity, leg length discrepancy, foot pronation and patellar hypermobility did not show any relationship with AKP. According to the systematic review by Waryasz and McDermott (2008), these factors are also considered as risk factors of AKP among runners. The absence of association in this study could be because of the small size of the sample. A bigger study in a large population may produce different results and may include other risk factors. Generalised ligamentous laxity, leg length discrepancy, foot pronation and patellar hypermobility problems may still need to be considered as part of a prehabilitation or rehabilitation programme. Therefore, proprioception and balance exercises could be recommended to reduce pain and improve functional status in patients with joint hypermobility (Brukner & Khan 2013; Halabchi et al. 2013; Witvrouw et al. 2000).

Leg length discrepancy also plays a role in the development of AKP (Halabchi et al. 2013). In the case of significant limb length discrepancy, correction of this problem is recommended, by ensuring that a shoe lift is not greater than one-half of the difference between the leg lengths (Halabchi et al. 2013). Excessive foot pronation is an intrinsic factor identified in some studies (Barton et al. 2010; Halabchi et al. 2013) and can be modified with custom-made foot orthoses after a careful foot assessment. Patellar hypermobility and patellar tilt are also potential risk factors for AKP (Halabchi et al. 2013; Witvrouw et al. 2000). Our study only showed that patellar tilt significantly contributed to AKP. As patellar tilt is usually a result of tight, deep, lateral retinacula structures, strapping in conjunction with a specific exercise programme is recommended for this problem.

The availability of rehabilitation services provided by a team of healthcare practitioners (e.g. physiotherapist, biokineticist and podiatrist) is important to prevent, treat and rehabilitate injuries. The outcome of this study is an indication of a need for prevention, treatment and rehabilitation of AKP injuries among runners. To provide a comprehensive programme, a multidimensional approach is required to cater for the various needs of the running population, especially from poorly resourced communities. A community-based approach could be considered to ensure that rehabilitation services are available, accessible and affordable to all runners who live in these communities. Decentralisation of rehabilitation services from urban to peri-urban and rural communities and task-shifting from qualified to less qualified rehabilitation personnel may need to be explored.

## Conclusion

This study showed a high prevalence of AKP and intrinsic modifiable factors that significantly contributed to AKP. These factors included quadriceps and gluteus muscle weakness, hamstring and iliotibial band tightness, and patellar malalignment. This study provides valuable baseline knowledge that can assist in the development of comprehensive rehabilitation programmes to manage AKP among runners. A community-based and multidimensional approach programme is recommended for athletes with AKP in poorly resourced communities.

## References

[CIT0001] Ageberg E., Zätterström R. & Moritz U., 1998, ‘Stabilometry and one – Leg hop test have high test – retest reliability’, *Scandinavian Journal of Medicine & Science in Sports* 8, 198–202. 10.1111/j.1600-0838.1998.tb00192.x 9764440

[CIT0002] Asayama I., Naito M., Fujisawa M. & Kambe T., 2002, ‘Relationship between radiographic measurements of reconstructed hip joint position and the Trendelenburg sign’, *Journal of Arthroplasty* 17, 747–751. 10.1054/arth.2002.33552 12216029

[CIT0003] Baker A.S. & Bitounis V.C., 1989, ‘Abductor function after total hip replacement. An electromyographic and clinical review’, *Journal of Bone and Joint Surgery [American]* 71, 47–50. 10.1302/0301-620X.71B1.2915004 2915004

[CIT0004] Barber T.C., Roger D.J., Goodman S.B. & Schurman D.J., 1996, ‘Early outcome of total hip arthroplasty using the direct lateral versus the posterior surgical approach’, *Orthopedics* 19, 873–875. 8905861 10.3928/0147-7447-19961001-11

[CIT0005] Barton C.J., Bonanno D., Levinger P. & Menz H.B., 2010, ‘Foot and ankle characteristics in patellofemoral pain syndrome: A case control and reliability study’, *Journal of Orthopdaedic & Sports Physical Therapy* 40, 286–296. 10.2519/jospt.2010.3227 20436240

[CIT0006] Bennell K.L., Talbot R.C., Wajswelner H., Techovanich W. & Kelly D., 1998 ‘Intra-rater and inter-rater reliability of a weight-bearing lunge measure of ankle dorsiflexion’, *Australian Journal of Physiotherapy* 44, 175–180. 10.1016/S0004-9514(14)60377-9 11676731

[CIT0007] Boling M., Padua D., Marshall S., Guskiewicz K., Pyne S. & Beutler A., 2010, ‘Gender differences in the incidence and prevalence of patellofemoral pain syndrome’, *Scandinavian Journal of Medicine & Science in Sports* 20(5), 725–730. 10.1111/j.1600-0838.2009.00996.x 19765240 PMC2895959

[CIT0008] Boyle K.L., Witt P. & Riegger-Krugh C., 2003, ‘Intrarater and interrater reliability of the Beighton and Horan Joint Mobility Index’, *Journal of Athletic Training* 38, 281–285. 14737208 PMC314385

[CIT0009] Brukner P. & Khan K.M., 2013, *Brukner & Khan’s clinical sports medicine*, 4th edn., chpt 33, pp. 684–714, McGraw-Hill Education (Australia) Pty Ltd, Sydney.

[CIT0010] Cichanowski H.R., Schmitt J.S., Johnson R.J. & Niemuth P.E., 2007, ‘Hip strength in collegiate female athletes with patellofemoral pain’, *Medicine and Science in Sports & Exercise* 39(8), 1227–1232. 10.1249/mss.0b013e3180601109 17762354

[CIT0011] Cook C., Hegedus E., Hawkins R., Scovell F. & Wyland D., 2010, ‘Diagnostic accuracy and association to disability of clinical test findings associated with patellofemoral pain syndrome’, *Physiotherapy Canada* 62(1), 17–24. 10.3138/physio.62.1.17 21197175 PMC2841549

[CIT0012] Cheung R., Zhang Z. & Ngai S., 2013, ‘Different relationships between the level of patellofemoral pain and quality of life in professional and amateur athletes’, *American Academy of Physical Medicine and Rehabilitation* 5, 568–572. 10.1016/j.pmrj.2012.12.007 23375635

[CIT0013] Dixit S., DiFiori J.P., Burton M. & Mines B., 2007, ‘Management of patellofemoral pain syndrome’, *Am Fam Physician* 75, 194–202. 17263214

[CIT0014] Downing N.D., Clark D.I., Hutchinson J.W., Colclough K. & Howard P.W., 2001, ‘Hip abductor strength following total hip arthroplasty. A prospective comparison of the posterior and lateral approach in 100 patients’, *Acta Orthopaedica Scandinavia* 72, 215–220. 10.1080/00016470152846501 11480593

[CIT0015] Finch C., Mahoney M., Townsend M. & Zazryn T., 2008, ‘Rural sports and recreational injuries in Australia: What do we know?’ *Australian Journal of Rural Health* 11, 151–158. 12950399

[CIT0016] Gabbe B., Bennell K., Wajswelnerc H. & Finch C., 2004, ‘Reliability of common lower extremity musculoskeletal screening tests’, *Physical Therapy in Sport* 5, 90–97. 10.1016/S1466-853X(04)00022-7

[CIT0017] Gogia P.P. & Braatz J.H., 1986, ‘Validity and reliability of leg length measurements’, *Journal of Orthopaedic & Sports Physical Therapy* 8, 185–188. 10.2519/jospt.1986.8.4.185 18802226

[CIT0018] Golle K., Granacher U., Hoffmann M., Wick D. & Muehlbauer T., 2014, ‘Effect of living area and sports club participation on physical fitness in children: A 4 year longitudinal study’, *BMC Public Health* 14, 499 https://doi:10.1186/1471-2458-14-499 24886425 10.1186/1471-2458-14-499 PMC4049502

[CIT0019] Halabchi F., Mazaheri R. & Seif-Barghi T., 2013, ‘Patellofemoral pain syndrome and modifiable intrinsic risk factors; how to assess and address?’, *Asian Journal of Sports Medicine* 4(2), 85 10.5812/asjsm.34488 23802050 PMC3690728

[CIT0020] Hoyle D., Latour M. & Bohannon R., 1991, ‘Intraexaminer, interexaminer, and interdevice comparability of leg length measurements obtained with measuring tape and metrecom’, *Journal of Orthopaedic & Sports Physical Therapy* 14, 263–268. 10.2519/jospt.1991.14.6.263 18796809

[CIT0021] Hudson Z. & Darthuy E., 2009 ‘Iliotibial band tightness and patellofemoral pain syndrome: A case-control study’, *Manual Therapy* 14(2), 147–151. 10.1016/j.math.2007.12.009 18313972

[CIT0022] Hunter D.J., Zhang Y.Q., Niu J.B., Felson D.T., Kwoh K., Newman A. et al., 2007, ‘Patella malalignment, pain and patellofemoral progression: The Health ABC Study, *Osteoarthritis and Cartilage* 15(10), 1120–1127. https://doi:10.1016/j.joca.2007.03.020 17502158 10.1016/j.joca.2007.03.020 PMC2042530

[CIT0023] Inan M., Alkan A., Harma A. & Ertem K., 2005, ‘Evaluation of the gluteus medius muscle after a pelvic support osteotomy to treat congenital dislocation of the hip’, *Journal of Bone and Joint Surgery [American]* 87, 2246–2252. 10.2106/JBJS.D.02727 16203890

[CIT0024] Ireland M.L., Willson J.D., Ballantyne B.T. & Davis I.M., 2003, ‘Hip strength in females with and without patellofemoral pain’, *Journal of Orthopaedic & Sports Physical Therapy* 33(11), 671–676. 10.2519/jospt.2003.33.11.671 14669962

[CIT0025] Kujala U.M., Jaakkola L.H., Koskinen S.K., Taimela S., Hurme M. & Nelimarkka O., 1993, ‘Scoring of patellofemoral disorders’, *Arthroscopy* 9, 159–163. 8461073 10.1016/s0749-8063(05)80366-4

[CIT0026] Melchione W.E. & Sullivan M.S., 1993, ‘Reliability of measurements obtained by use of an instrument designed to indirectly measure iliotibial band length’, *Journal of Orthopaedic & Sports Physical Therapy* 18, 511–515. 10.2519/jospt.1993.18.3.511 8298633

[CIT0027] Pai V.S., 1996, ‘Significance of the trendelenburg test in total hip arthroplasty’, *Journal of Arthroplasty* 11, 174–179. 10.1016/S0883-5403(05)80013-0 8648312

[CIT0028] Patel D.R. & Villalobos A., 2017, ‘Evaluation and management of knee pain in young athletes: Overuse injuries of the knee’, *Translation Pediatrics* 6(3), 190–198. https://doi.org/10.21037/tp.2017.04.05 10.21037/tp.2017.04.05 PMC5532199 28795010

[CIT0029] Piva S.R., Goodnite E.A. & Childs J.D., 2005, ‘Strength around the hip and flexibility of soft tissues in individuals with and without patellofemoral pain syndrome’, *Journal of Orthopaedic & Sports Physical Therapy* 35, 793–801. 10.2519/jospt.2005.35.12.793 16848100

[CIT0030] Puniello M.S., 1993, ‘Iliotibial band tightness and medial patellar glide in patients with patellofemoral dysfunction’, *Journal of Orthopaedic & Sports Physical Therapy* 17, 144–148. 10.2519/jospt.1993.17.3.144 8472078

[CIT0031] Ramesh M., O’Byrne J.M., McCarthy N., Jarvis A., Mahalingham K. & Cashman W.F., 1996, ‘Damage to the superior gluteal nerve after the Hardinge approach to the hip’, *Journal of Bone and Joint Surgery [British]* 78, 903–906. 10.1302/0301-620X78B6.1289 8951004

[CIT0032] Redmond A., Crosbie J. & Ouvrier R., 2006, ‘Development and validation of a novel rating system for scoring standing foot posture: The foot posture index’, *Clinical Biomechanics* 21, 89–98. 10.1016/j.clinbiomech.2005.08.002 16182419

[CIT0033] Reikeraas O., Lereim P., Gabor I., Gunderson R. & Bjerkreim I., 1996, ‘Femoral shortening in total hip arthroplasty for completely dislocated hips’, *Acta Orthopaedica Scandinavia* 67, 33–36. 10.3109/17453679608995605 8615099

[CIT0034] Singer B. & Singer K., 2009, ‘Anterior knee pain scale’, *Australian Journal of Physiotherapy* 55(2), 140 10.1016/S0004-9514(09)70048-0 19534016

[CIT0035] Tria A.J., Palumbo R.C. & Alicea J.A., 1992, ‘Conservative care for patellofemoral pain’, *Orthopaedic Clinics of North America* 23(4), 545–554. 1408039

[CIT0036] Van Gent R.N., Siem D., Van Middelkoop M., Van Os A.G., Bierma-Zeinstra S.M.A. & Koes B.W., 2007, ‘Incidence and determinants of lower extremity running injuries in long distance runners: A systematic review’, *British Journal of Sports Medicine* 41(8), 469–480. 10.1136/bjsm.2006.033548 17473005 PMC2465455

[CIT0037] Watson C.J., Leddy H.M., Dynjan T.D. & Parham J.L., 2001, ‘Reliability of the lateral pull test and tilt test to assess patellar alignment in subjects with symptomatic knees: Student raters’, *Journal of Orthopaedic & Sports Physical Therapy* 31, 368–374. 10.2519/jospt.2001.31.7.368 11451307

[CIT0038] Waryasz G.R. & McDermott A.Y., 2008, ‘Patellofemoral pain syndrome: A systematic review of anatomy and potential risk factors’, *Dynamic Medicine* 7, 9 10.1186/1476-5918-7-9 18582383 PMC2443365

[CIT0039] Witvrouw E., Lysens R., Bellemans J., Cambier D. & Vanderstraeten G., 2000, ‘Intrinsic risk factors for the development of anterior knee pain in an athletic population’, *The American Journal of Sports Medicine* 28(4), 480–489. 10.1177/03635465000280040701 10921638

